# Adaptation of the Emotional Contagion Scale (ECS) and gender differences within the Greek cultural context

**DOI:** 10.1186/1744-859X-7-14

**Published:** 2008-08-21

**Authors:** Pantelis Kevrekidis, Petros Skapinakis, Dimitris Damigos, Venetsanos Mavreas

**Affiliations:** 1 Department of Psychiatry, University of Ioannina School of Medicine, Ioannina, 45110, Greece

## Abstract

**Background:**

The Emotional Contagion Scale (ECS) is a self-report scale used to measure individual differences in susceptibility to converge towards the emotions expressed by others. The main aim of the present paper was to examine the psychometric properties of the Greek translation of the scale.

**Methods:**

The Greek ECS was completed by 691 undergraduate students (312 males and 379 females). To investigate the factor structure of the ECS, principal components analysis (PCA) was used.

**Results:**

The results showed that a four-factor model was tenable. Regarding homogeneity, the Greek ECS version showed acceptable results for the full scale (α = 0.74) but not for all subscales. Gender differences were also identified concerning the susceptibility to emotional contagion between men and women. Women score significantly higher than men for all the different emotions described by the ECS (love, happiness, sadness) except the anger emotion, where there was no significant difference.

**Conclusion:**

The Greek version of the ECS showed good psychometric properties. It can be used to assess susceptibility to emotional contagion in correlation with psychopathological processes, mood and anxiety disorders primarily. The usefulness of the ECS in the fields of group psychotherapy and health psychology is also under consideration. Further investigation is needed in all these areas.

## Background

The term 'emotional contagion' refers to the tendency one has to 'catch' another person's emotions [1]. According to Hatfield *et al*. [1], this includes the tendency to convert emotionally to each other, by mimicking and synchronising with the facial, postural and instrumental expressions of the other party.

It is postulated that emotional contagion operates continuously and non-consciously through different non-verbal communicative channels documented in body language [2], in vocal expressions [3], and in facial expressions [4].

From a clinical perspective, emotional contagion has been shown to be a useful concept in studies concerning mood and anxiety disorders [5], psychotherapy [6] and health psychology [7-10].

### Description of Emotional Contagion Scale (ECS)

Despite the growing interest in emotional contagion theory, until recently there were no assessment tools to measure the phenomenon. The main goal was to develop a short and reliable instrument to measure individual differences to emotional contagion. The first, psychometrically evaluated questionnaire was developed by Doherty *et al. *[11], which was revised twice from a 38-item questionnaire to a 18-item version, and finally to a 15-item version. This scale proved to have high reliability (Cronbach α = 0.90). Although the original ECS is presented as a one-factor solution, a multidimensional solution is also suggested [12]. The ECS is the only self-reported scale that measures the susceptibility to emotional contagion in cross-culturally relevant contexts. It includes the five basic emotions of love, happiness, anger, fear and sadness. Regarding gender differences, it has been consistently reported that women rate themselves as more susceptible to emotional contagion compared to men [11-13].

Until now, there has been no reliable and valid instrument in Greek to measure susceptibility to emotional contagion. The aim of the present study was therefore to adapt the ECS to the Greek cultural context and to explore its psychometric properties. A secondary aim was to investigate possible gender differences concerning the susceptibility to emotional contagion within this cultural context.

## Materials and methods

### Participants and procedures

A total of 703 questionnaires were administered to undergraduate University students; 691 questionnaires were valid (98.3%). The sample consisted of 379 women with a mean age of 19.9 years (standard deviation (SD) = 3.28 years) and 312 men with a mean age of 20.76 years (SD = 3.50 years). The age span for both men and women was 18 to 45 years. The sample participated voluntarily and the ECS was completed after standardized instructions were given.

The ECS is a 15-item self-reported scale, which assesses the susceptibility to 'catch' the emotions expressed by others. The ECS consists of five basic emotions: love, happiness, sadness, anger and fear. Each emotion is represented by three items that are scored on a 5-point Likert scales from not at all (1) to always (5). The entire ECS scale takes no more than 5 minutes to administer.

The ECS questionnaire was translated from English to Greek independently by the author and another professional translator and then the Greek text was back-translated to English by a bilingual person for crosschecking. The translations were compared, and the few discrepancies found consisted of different choices of synonymous words; the structure or the meaning of the sentences was not changed (see Additional file 1).

### Data analysis

Descriptive statistics and principal component analysis (PCA) were conducted using SPSS v. 14.0 (SPSS Inc., Chicago, IL, USA). Psychometric evaluation of the ECS scale and its subscales were assessed with the Cronbach α [14], using the α > 0.70 criterion for adequate homogeneity [15]. We also applied t tests in order to detect possible gender differences in susceptibility to emotional contagion.

## Results

### PCA and internal consistency of the ECS

The 15 items of the ECS scale were subjected to PCA. Both varimax and oblimin oblique rotations were conducted. Prior to performing PCA, the suitability of data for factor analysis was assessed. Inspection of the correlation matrix revealed the presence of many coefficients of 0.3 and above. The Kaiser-Meyer-Oklin value was 0.80, exceeding the recommended value of 0.6 [16,17] and the Bartlett test of sphericity [18] reached statistical significance, supporting the factorability of the correlation matrix (x^2 ^= 2028.4, df = 105, p < 0.0001).

**Figure 1:**
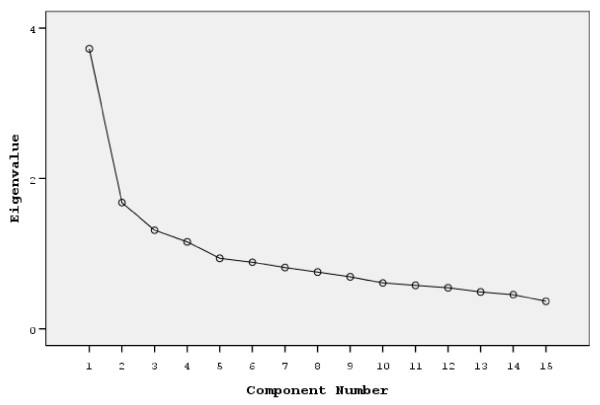
**Screeplot of Emotional Contagion Scale (ECS), 15 item version**. Screeplot in the 15-item ECS shows a clear cut after the second component.

Principal components analysis revealed the presence of four components with eigenvalues exceeding 1, explaining 24.91%, 11.20%, 8.8%, and 7.63% of the variance respectively [19,20]. An inspection of the screeplot revealed a clear break after the second component (Figure 1). However, using Catell's screeplot [21], it was decided to retain four components for further investigation because their eigenvalue was >1, plus a fifth component because its eigenvalue was close to 1.

To aid in the interpretation of these five components, varimax rotation was performed. The rotated solution revealed the presence of multidimensional structure, with two 'clear' components showing strong loadings: (a) love items 6, 9, 12 with factor loadings ranging from 0.73 to 0.83 and Mload = 0.80 and (b) happiness items 2, 3, 11 with loadings ranging from 0.63 to 0.78 and Mload = 0.72. However, the three remaining components did not show clear-cut factor loadings. For example, in component 2, there are strong loadings on item 7 (anger item with loading 0.72), item 10 (anger item with loading 0.719) and the next strong loading is on item 13 (fear item according to the constructor of the ECS scale with loading 0.681) (Table 1). The same applies as far as factor loadings are concerned to the other two components (3 and 5) as well as to the varimax rotations performed for men and women separately.

**Table 1:** Emotional Contagion Scale (ECS) factor loadings with 15 items

**Emotion**	**Item**	**Components**				
		
		**1**	**2**	**3**	**4**	**5**
Love	6	0.732	0.153	0.019	0.177	0.083
Love	9	0.833	0.021	0.205	0.100	0.018
Love	12	0.820	0.024	0.114	0.153	0.007
Happiness	2	0.056	-0.001	0.058	0.788	-0.037
Happiness	3	0.184	0.103	0.179	0.632	0.193
Happiness	11	0.214	0.164	0.036	0.767	-0.030
Fear	8	0.073	0.122	0.086	0.038	0.920
Fear	13	0.110	0.681	0.224	0.116	0.068
Fear	15	0.155	0.225	0.542	-0.002	-0.180
Anger	5	-0.031	0.558	0.068	-0.009	0.141
Anger	7	0.038	0.720	0.009	0.191	-0.012
Anger	10	0.116	0.719	0.149	-0.003	-0.024
Sadness	1	-0.011	0.146	0.764	0.116	0.083
Sadness	4	0.100	0.205	0.470	0.193	0.277
Sadness	14	0.160	-0.012	0.803	0.048	0.086

Because of the fact of loadings on different items, it was decided to remove items 8, 13, and 15, which constitute the fear items and seemed to be dispersed in different components (namely components 2, 3 and 5), and re-perform factor analysis (PCA) with items 8, 13 and 15 (fear) excluded.

Principal components analysis of the 12 items (fear items 8, 13, and 15 excluded) revealed the presence of four components with eigenvalues exceeding 1, explaining 23.31%, 12.67%, 10.52%, and 9.43% of the variance, respectively.

**Figure 2:**
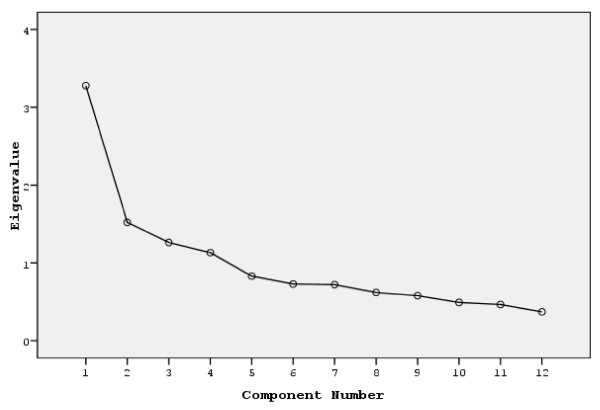
**Screeplot of Emotional Contagion Scale (ECS), 12 item version**. Screeplot in the 12-item ECS shows a clear cut after the second component.

An inspection of the screeplot revealed a clear cut after the second component (Figure 2). Using Cattel's screeplot it was decided to retain four components for further investigation. To aid the interpretation of these four components, oblimin oblique rotation was performed (Table 2). The rotated solution revealed the presence of a multidimensional structure with four components, with the first component including the love items (6, 9, 12), the second component including happiness items (2, 3, 11), the third component including the sadness items (1, 4, 14), and the fourth component including anger items (5, 7, 10). Thus, the interpretation of the four components solution yields a four-factor model.

**Table 2:** Emotional Contagion Scale (ECS) factor loadings with 12 items: oblimin oblique rotation

**Emotion**	**Item**	**Components**			
		
		**1**	**2**	**3**	**4**
Love	9	0.863			
Love	12	0.855			
Love	6	0.736			
Happiness	2		0.837		
Happiness	11		0.782		
Happiness	3		0.629		
Sadness	14			0.837	
Sadness	1			0.830	
Sadness	4			0.530	
Anger	7				0.746
Anger	10				0.717
Anger	5				0.677

The same four factor model applies to men (n = 312) and women (n = 379) of the sample separately. The component loadings in the oblimin oblique rotation ranged from 0.53 to 0.86 (Mload = 0.75).

The internal consistency for the full ECS was acceptable, (Cronbach α = 0.74). For the internal consistency for each factor alone, the Cronbach α > 0.70 was met only by the love factor (3 items) (Table 3).

**Table 3:** Mean scores and internal consistency of Emotional Contagion Scale (ECS)

		**Total (n = 691)**		**Males (n = 312)**		**Females (n = 379)**		
**Scales**	**No. of items**	**M**	**SD**	**M**	**SD**	**M**	**SD**	**Cronbach α**

Full ECS	12	3.31	0.55	3.12	0.55	3.46**	0.50	0.74
Love	3	3.86	0.86	3.65	0.90	4.04**	0.79	0.76
Happiness	3	3.82	0.78	3.68	0.82	3.94**	0.73	0.64
Sadness	3	2.87	0.83	2.47	0.74	3.19**	0.77	0.61
Anger	3	2.68	0.79	2.69	0.79	2.67	0.79	0.53

Mean scores for the females significantly larger than the mean scores for the males by * p < 0.05, ** p < 0.01

### Gender differences

Analyses by t test revealed the presence of gender differences in the susceptibility to emotional contagion and this concerns the full ECS questionnaire. Women score higher than men to all affect factors (love items 6, 9, 12, happiness items, 2, 3, 11, sadness items, 1, 4, 14) but not to the anger affect factor (items 5, 7, 10) where there is not significant difference between men and women (Table 3).

## Discussion

The main purpose of the present study was to adapt the ECS scale to the Greek cultural context, to define its factor structure within this context, and secondly to investigate possible gender differences regarding emotional contagion.

PCA revealed four components, the loadings of which met the generally adopted criteria for minimal loading levels [22]. The rotated solution revealed a four-factor structure, which is theoretically and statistically justified [13]. These four factors represent the four subscales of the ECS scale namely: love, happiness, sadness, and anger. The internal consistency for the full ECS scale is acceptable, however, the internal consistency criterion (Cronbach α > 0.70) is not met for each subscale, probably because of the low number of items for each emotion. Thus, one should avoid selective administration of the subscales as this could lead to erroneous conclusions [23].

### Comparison with previous studies

The Swedish adaptation of the ECS [13] was taken into consideration because of its comprehensive data analysis. The item loadings of the present study were to a large extent concordant with those reported in the Swedish adaptation of the ECS in the oblimin oblique rotation (0.60 to 0.85, (Mload = 0.77)). The internal consistency for the full ECS in the present study (Cronbach α = 0.74), was somewhat lower than the Swedish version (Cronbach α = 0.76) and even lower than the original American version (Cronbach α = 0.90). While the American version of the ECS is one-dimensional, both the Greek and Swedish versions are multidimensional as a result of factor analysis, which is also referred as being applicable in the American study [12].

The findings of this study do replicate the findings of the Swedish study [13]. Women score higher than men and are more susceptible to emotional contagion for three of the basic emotions, namely love, happiness, and sadness, but not the anger. Further research is needed to explore this phenomenon. Gender differences must be taken into account during assessment of the susceptibility of the general population to emotional contagion.

The major advantage of the ECS scale compared to other empathy scales is that the ECS provides information that others do not. Mehrabian and Epstein [24] scale is widely used to measure vicarious responding and arousability. Both scales provide information about emotional arousal, but the ECS is the only one that reports the congruence between the emotional stimulus and the emotional response. The emotion experienced by an individual is in direct correspondence with the emotion observed, and this refers mostly to the primitive emotional contagion.

### Practical uses of the ECS scale

There is some evidence that patients with antisocial personality disorder have difficulties in processing non-verbal emotional stimuli [25]. These findings suggest that antisocial personality disorder subjects may exhibit difficulties in expressing emotional contagion. This perhaps implies that the ECS could be a potential instrument in the assessment of the lack of emotional contagion associated with this personality disorder. However, this needs to be tested in clinical settings.

Another area where the ECS could probably have a potential value is the area of developmental disorders. Autistic adolescents who belong to the high susceptibility group for emotional contagion may likely have another prognosis compared to those of low susceptibility. Thus, the ECS could theoretically be used in the assessment of developing social skills in autism and related conditions [26]. Clinical research must be conducted to yield empirical data in this area.

ECS could potentially be useful in schizophrenia research [27]. There is evidence, for example, that patients with schizophrenia exhibit greater skin conductance reactivity compared to controls when viewing emotional films, but are less facially expressive than controls and report experiences of both positive and negative emotions [28]. It would probably be of interest to investigate whether there are subgroups of patients with schizophrenia showing low or high susceptibility to emotional contagion.

Studies examining the application of scales measuring the emotional contagion in situations like depression and burnout among health professionals are also of interest. According to some research, these scales may be useful tools for the identification, prevention and management of professionals at risk for mood and anxiety disorders [29,30]. Finally, the ECS could probably be used as a tool addressing emotional contagion in health psychology [7-10], and psychotherapy [6,31-33].

Further studies must be conducted, with the aim of investigating the practical and clinical as well as theoretical implications of susceptibility to different levels of emotional contagion.

### Limitations of the present study

The findings of this study should be considered in the context of the following limitations: (1) we only studied undergraduate students and the generalisability of our results to other subjects of different age or education may not be possible. In addition, the psychometric properties of the scale may differ in clinical settings. In all these settings, future investigators should try to verify the factor structure of the scale. (2) The small number of items per emotion (three items) may have compromised the factor structure of the scale. By contrast, the few items facilitate the data collection. (3) Finally, although the ECS is grounded on a good theoretical basis, there are very few data to empirically support its usefulness in clinical settings.

## Conclusion

The findings of the present study suggest that the Greek version of the ECS is acceptable and it is in concordance with both the American and Swedish versions of the ECS. It might be used in clinical settings to assess susceptibility to emotional contagion in correlation with psychopathological processes in mood and anxiety disorders, personality disorders, psychosis, and autism spectrum disorders.

## Competing interests

The authors declare that they have no competing interests.

## Authors' contributions

PK conceived the idea and design of the study, carried out the data collection and data analysis, drafted the manuscript and helped in the interpretation of the results. PS critically revised the article and helped in data analysis and interpretation of results. DD and VM helped in the design of the study and interpretation of results. All authors approved the final version of the manuscript.

## Supplementary Material

Additional file 1 **The Greek version of the Emotional Contagion Scale and the original American version**. The Greek version is differentiated from the American version by data analysis. Thus, three items (8, 13, 15) from the original ECS were excluded in the Greek ECS. Click here for file
