# Supplementary material for: Adaptation of the Emotional Contagion Scale (ECS) and gender differences within the Greek cultural context
Source: Ann Gen Psychiatry. 2008 Aug 21;7:14. doi: 10.1186/1744-859X-7-14 (PMC2533309; doi:10.1186/1744-859X-7-14)
Supplement: Additional file 1 — The Greek version of the Emotional Contagion Scale and the original American version. The Greek version is differentiated from the American version by data analysis. Thus, three items (8, 13, 15) from the original ECS were excluded in the Greek ECS. [file 1744-859X-7-14-S1.doc]

Το ερωτηματολόγιο αυτό αποτελείται από 15 προτάσεις που αφορούν τον ΔΙΚΟ ΣΟΥ τρόπο να σκέφτεσαι, να αισθάνεσαι και να ενεργείς σε διάφορες καταστάσεις. Κάθε πρόταση έχει 5 διαβαθμίσεις από το ποτέ (1) έως το πάντα (5). Σημείωσε τον αριθμό που εσύ θεωρείς ότι είναι ο σωστός για σένα.

(1) ποτέ (2) μερικές φορές (3) συχνά (4) αρκετά συχνά (5) πάντα

| ποτέ | |  | πάντα | | | |
| --- | --- | --- | --- | --- | --- | --- |
| 1. Εάν ο συνομιλητής μου αρχίζει να κλαίει, τότε μου έρχονται δάκρυα στα μάτια. | 1 | 2 | 3 | 4 | 5 |  |
| 2. Το να είμαι με κάποιον που είναι χαρούμενος, με κάνει να αισθάνομαι καλύτερα,  όταν είμαι στεναχωρημένος. | 1 | 2 | 3 | 4 | 5 |  |
| 3. Όταν κάποιος μου χαμογελά εγκάρδια, ανταποδίδω το χαμόγελό του και αισθάνομαι όμορφα μέσα μου. | 1 | 2 | 3 | 4 | 5 |  |
| 4. Γεμίζω με συναισθήματα λύπης όταν κάποιος μου διηγείται τον θάνατο κάποιου αγαπημένου του προσώπου. | 1 | 2 | 3 | 4 | 5 |  |
| 5. Σφίγγω τα δόντια μου και τους ώμους μου, όταν βλέπω θυμωμένα πρόσωπα στις ειδήσεις. | 1 | 2 | 3 | 4 | 5 |  |
| 6. Όταν κοιτάζω μέσα στα μάτια τους αγαπημένους μου, καταλαμβάνομαι από ρομαντικές σκέψεις. | 1 | 2 | 3 | 4 | 5 |  |
| 7. Εκνευρίζομαι όταν βρίσκομαι μεταξύ θυμωμένων ανθρώπων. | 1 | 2 | 3 | 4 | 5 |  |
| 8. Όταν βλέπω τα φοβισμένα πρόσωπα των θυμάτων στις ειδήσεις, προσπαθώ να φανταστώ το πώς αισθάνονται. **[excluded due to data analysis]** | 1 | 2 | 3 | 4 | 5 |  |
| 9. «Λιώνω», όταν αυτός/ ή που αγαπώ με αγκαλιάζει. | 1 | 2 | 3 | 4 | 5 |  |
| 10. Φορτίζομαι όταν τυχαίνει να ακούσω έναν καβγά. | 1 | 2 | 3 | 4 | 5 |  |
| 11. Όταν περιτριγυρίζομαι από χαρούμενους ανθρώπους, γεμίζω με χαρούμενες σκέψεις. | 1 | 2 | 3 | 4 | 5 |  |
| 12. Το αισθάνομαι σε όλο το σώμα, όταν ο/ η αγαπημένος/ η μου με αγγίζει. | 1 | 2 | 3 | 4 | 5 |  |
| 13. Καταλαβαίνω ότι φορτίζομαι όταν βρίσκομαι μεταξύ ανήσυχων ανθρώπων.  **[excluded due to data analysis]** | 1 | 2 | 3 | 4 | 5 |  |
| 14. Κλαίω, όταν βλέπω λυπητερά έργα. | 1 | 2 | 3 | 4 | 5 |  |
| 15. Εάν τύχει να ακούσω την κραυγή ενός τρομαγμένου παιδιού, στην αίθουσα αναμονής του οδοντιατρείου, γίνομαι νευρικός/ ή. **[excluded due to data analysis]** | 1 | 2 | 3 | 4 | 5 |  |

The Emotional Contagion Scale

| never | |  | always | | | |
| --- | --- | --- | --- | --- | --- | --- |
| 1. If someone I’m talking with begins to cry, I get teary-eyed. | 1 | 2 | 3 | 4 | 5 |  |
| 2. Being with a happy person picks me up when I’m feeling down. | 1 | 2 | 3 | 4 | 5 |  |
| 3. When someone smiles warmly at me, I smile back and feel warm inside. | 1 | 2 | 3 | 4 | 5 |  |
| 4. I get filled with sorrow when people talk about the death of their loved ones. | 1 | 2 | 3 | 4 | 5 |  |
| 5. I clench my jaws and my shoulders get tight when I see the angry faces on the news. | 1 | 2 | 3 | 4 | 5 |  |
| 6. When I look in the eyes of the one I love, my mind is filled with thoughts of romance. | 1 | 2 | 3 | 4 | 5 |  |
| 7. It irritates me to be around angry people. | 1 | 2 | 3 | 4 | 5 |  |
| 8. Watching the fearful faces of victims on the news makes me try to imagine how they might be feeling. | 1 | 2 | 3 | 4 | 5 |  |
| 9. I melt when the one I love holds me close. | 1 | 2 | 3 | 4 | 5 |  |
| 10. I tense when overhearing an angry quarrel. | 1 | 2 | 3 | 4 | 5 |  |
| 11. Being around happy people fills me with happy thoughts. | 1 | 2 | 3 | 4 | 5 |  |
| 12. I sense my body responding when the one I love touches me. | 1 | 2 | 3 | 4 | 5 |  |
| 13. I notice myself getting tense when I’m around people who are stressed out. | 1 | 2 | 3 | 4 | 5 |  |
| 14. I cry at sad movies. | 1 | 2 | 3 | 4 | 5 |  |
| 15. Listening to the shrill screams of a terrified child in a dentist’s waiting room makes me feel nervous. | 1 | 2 | 3 | 4 | 5 |  |

Doherty, R. W. (1997). The emotional contagion scale: A measure of individual differences. *Journal of Nonverbal Behavior, 21*(2), 131-154.
